# Does electronic data collection perform better than paper-based data collection in health research fieldwork? A participatory action research in Zanzibar

**DOI:** 10.1136/bmjph-2023-000749

**Published:** 2024-06-03

**Authors:** Omar Juma Othman, Eden Mashayo, Jamison Jones, Kajal Shah, Christine Graham, Ai Chee Yong, Ronnie Graham, Fatma Omar, Ving Fai Chan

**Affiliations:** 1 Zanzibar Ministry of Health, Zanzibar, United Republic of Tanzania; 2 Vision Care Foundation, Dar-es-Salaam, United Republic of Tanzania; 3 Loyola University Chicago, Chicago, Illinois, USA; 4 Dublin Institute of Technology, Dublin, Ireland; 5 Centre for Public Health, Queen's University Belfast, Belfast, UK; 6 Vision Action, London, UK; 7 College of Health Sciences, University of KwaZulu-Natal, Durban, South Africa

**Keywords:** Public Health, Community Health, Prevalence

## Abstract

**Introduction:**

Technological advancement in low-resource settings is opening the gateway to implementation of electronic data collection methods that improve data quality. We examined the concerns to use electronic data collection tool in Zanzibar, codeveloped a tool that addressed the concerns and evaluated the process and limitations of incorporating an electronic data collection tool aside from paper-based during a community-based study in Zanzibar.

**Methods:**

The science of improvement Plan-Do-Study-Act model guided this mixed-method participatory action research (PAR). From November 2022 to October 2023, 14 data collection team members participated in (1) a consultative workshop with a fishbone analysis to understand their hesitance to use electronic data collection tools for fieldwork (Plan); (2) developing implementation and evaluation plan for the paper-based method (Do); (3) assessing the proportion of errors and challenges faced using paper-based method (Study); and (4) codeveloping, implementing and assessing an electronic data collection tool (Act).

**Results:**

Stakeholders were hesitant to use electronic data collection tools because of fear of lost data due to poor internet, insufficient competency with technology due to lack of training, unfamiliarity with technology in general and fear of lost wages. The study revealed that using a paper-based data collection tool during baseline was time-consuming, with 12.8% of responses being errors (2611 errors out of 20 398 responses). However, once implemented, the electronic data collection application was fast and simple, with minimal errors (0.02%). Overall, there is a need to improve devices’ storage capacity devices and provide more training.

**Conclusion:**

Using the PAR approach, we understood the concerns with electronic data collection tools, allowed the team to experience the challenges faced with the paper-based collection method, codeveloped an appropriate solution and changed their attitude towards using technology that could increase accuracy and efficiency of their fieldwork.

**WHAT IS ALREADY KNOWN ON THIS TOPIC**

- Electronic data collection methods are gaining traction for gathering trial and surveillance data in low/middle-income countries (LMICs). However, their adoption for health research fieldwork in LMICs remains limited, including Zanzibar.

**WHAT THIS STUDY ADDS**

- This study shows that by involving the data collection team in identifying the reservation in using electronic data collection methods, critically appraising the conventional paper-based methods, creating and assessing the solution, we could help participants to develop new skills and knowledge, and also boost their confidence and sense of agency.

**HOW THIS STUDY MIGHT AFFECT RESEARCH, PRACTICE OR POLICY**

- The lessons learnt from this participatory action research could contribute to the knowledge pool to inspire researchers in other health sectors to reassess their feasibility in using electronic data collection methods that could improve data collection quality and efficiency.

## Introduction

 Public health research studies are important to understand the population’s health and the factors that affect it.^1^,^3^ These factors, more commonly known as determinants of health, include biological, behavioural, social and environmental.^1^,^3^ Often multidisciplinary, public health research studies require relatively large sample sizes to provide more reliable results with smaller error margins and lower deviation standards.^1^,^3^ Furthermore, depending on the design of the studies, data collection methods could be complex and for a significant duration.^1^,^3^ These processes called for effective data collection tools.

Traditionally, data from field studies are collected using the paper-based format, and then data were checked for inconsistencies, followed by capturing them onto an electronic database, and cleaned before being analysed.^4^ These multiple layers of data transmission are prone to human errors. When these errors are systematic, the studies will produce inaccurate findings, interpretations and conclusions. Hence, electronic data collection becomes an alternative in many medium-scale to large-scale studies to minimise human errors in the data collection process.^5 6^

Electronic data collection tools allow large-volume data storage.^7^ This data protection through encryption and quick and safe data transfer could then be processed, reviewed and analysed efficiently.^7 8^ With accessibility to smartphone subscriptions reaching 97% globally and 70% in sub-Saharan Africa^9^ and the advancement in computer technology, public health research studies could benefit from using electronic data collection tools. While electronic data collection tools have been widely used in high-resource settings in clinical studies,^8 10 11^ their use, feasibility, acceptability and operational challenges have been under-studied in low-resource settings.

A study in Zanzibar^12^ and Ethiopia^13^ a decade ago showed that electronic data entry was faster, cheaper and more accurate than paper data collection in large-scale field survey, but anecdotal evidence still shows high hesitancy to using electronic data collection tool in field research in Zanzibar. In response to these knowledge gaps, we aim to use the field experience from a longitudinal study called Women’s Empowerment through Investing in Zanzibari Craftswomen’s Eyesight (WE-ZACE) to understand the reservations that the local implementation teams had about using electronic data collection tools, codevelop a need-based electronic data collection tool with local stakeholders and compare the data collectors’ performance and experience using paper-based at baseline, and electronic data collection tools at endline.

## Methods

### Patient and public involvement

This participatory cction research (PAR) is part of the WE-ZACE patient and public involvement strategy. As described in this article, the local data collection team were invited to identify the problems, appraise the conventional paper-based methods and cocreating and assessing the solution.

### Research design

Our team used a mixed-method PAR apporach^14^ in this WE-ZACE substudy. The WE-ZACE project was conducted in Zanzibar among craftswomen 40 years and older. The project aims to understand the relationship between correcting age-related near vision impairment (presbyopia) among craftswomen and their economic and social empowerment by following the craftswomen for 6 months after they were provided with near-vision spectacle correction. The WE-ZACE study allowed us to involve extensive engagement with the local data collection team in Zanzibar, leading to the codevelopment of an electronic data collection tool that addressed their unique needs and concerns.

The science of improvement: testing changes—model for improvement^15^ guided the design of our work as it involves a systematic process for data capture, analysis, methods adaptation and generating lessons that contribute to project improvement. Table 1 shows the model of improvement for our study.

**Table 1:** Model of improvement for the study

Questions	Answers
What are we trying to accomplish?	Achieve better data quality when collecting field surveys in a low-resource setting (Zanzibar)
How will we know a change is an improvement?	Fewer incomplete, missing and illogical dataReported positive experience by the data collection team
What change can we make that will result in improvement?	A well-designed, user-friendly electronic data collection toolEffective training of data collectors

The work consisted of four steps, adapted from Plan-Do-Study-Act (PDSA) cycles, based on action-based learning. These included Plan (November 2022), Do (February 2023), Study (April 2023 to October 2023) and Act (November 2023).

The Plan phase consisted of a 2-day consultative workshop where one of the objectives was to develop the study implementation plan for the WE-ZACE study. One session was dedicated to determining (1) the preferred approach for data collection and (2) the reasons for choosing the approach. The WE-ZACE data collection team members (n=8), eyecare personnel (n=4), a data capturer, and a statistician attended the workshop. Using the responses from the workshop, we conducted a fishbone analysis to determine the root cause of the unwillingness to use electronic data collection tools for fieldwork. The major bones highlight the primary reasons for hesitations regarding the use of electronic data collection tools, while the minor bones elaborate on the challenges associated with each key reason. As an illustration, one key reason identified was the fear of losing wages (major bone). This concern stemmed from the perception that the efficiency of electronic data collection tools, compared with paper-based methods, resulted in fewer working days (minor bone). Given that wage payment was based on a daily rate, this increased efficiency was perceived as a disadvantage, leading to reduced pay (minor bone). A subsequent meeting was held among the core project management team (chief investigator, principal investigator, lead statistician and implementation manager) to review the responses and design the implementation and evaluation plan for the data collection process.

This is followed by the Do phase. At baseline, four teams of data collectors, each consisting of one eyecare personnel and two data collectors, underwent a 5-day training to standardise the survey procedures and data recording using a paper-based recording form. The paper-based facilitated skipping options to relevant questions based on the respondent responses, ensuring the interview flow was directed appropriately. Each section was accompanied by a preamble that included instructions to help both the data collectors and respondents understand the process before proceeding. Instruction manual was provided as part of the training. A data capturer was trained to enter the data into an Excel spreadsheet and identify inconsistencies.

Subsequently, the baseline survey (Study) was conducted. On the first 2 days, on-the-spot supervision by team supervisors were conducted during interviews to ensure questions were asked correctly and provided additional information if needed for clarification. Subsequently, daily supervision was carried out throughout the data collection period to detect any inconsistencies in answers and logical incoherence. The lead statistician and his team of data capturers entered and checked the data records daily to detect any inconsistencies. Identifying errors or inconsistencies in the data involved reviewing the completed papers at the end of each day, and revisiting respondents to address blank questions or inconsistencies. The primary outcome for data quality was the number of errors detected from the data (this included incomplete or missing data, as well as illogical or inconsistent data).

During the final phase (Act), the lead statistician developed the electronic data collection tool specifically used for the WE-ZACE project data collection based on the Plan and Study phase findings. Kobo Collect, an open-source software tool, was chosen for the project as it was affordable and accessible to the team. The lead statistician downloaded the software and customised its use for the project. The software was compatible with Android devices and allowed offline data collection, which is particularly useful in remote areas in Zanzibar with limited internet connectivity. Questions were designed with skip logical and prompt questions to ensure minimal errors. It allowed supervision conducted in real-time where data could be immediately transmitted to the database after each interview, facilitating prompt error detection and correction. The data supervisory team identified potential issues such as free text, prescriptions findings and rectify errors during data collection. We also assured the data collectors that they would be paid a fixed monthly consultation wage, regardless of the early completion of their data collection.

In the endline, the same teams of data collectors underwent a 2-day training on how to use the electronic tool and an additional 2 days of field testing. During these 2 days, on-the-spot supervision by team supervisors were conducted during interviews to ensure questions were asked correctly and provided additional information if needed for clarification. The data supervisory team checked the data records daily to detect any inconsistencies. The primary outcome for data quality was the number of errors detected from the data (including incomplete, missing data and illogical data). We then conducted a 2-hour debriefing session with the data collection team to obtain feedback on their experience using the electronic data collection tool. The team was asked five questions:

- Can you tell me what worked very well during the data collection process for the WE-ZACE project, during baseline and endline? Give examples.
- Can you tell me what did not work well during the data collection process for the WE-ZACE project during baseline and endline? Give examples.
- Can you tell me if any changes were made to how you collected data based on your previous experience? Has it worked better? Give examples.
- How do you think we could have done better?
- Have you detected any differences between the endline survey’s data collection methods and the baseline survey? If so, what changed? Please explain.

Their responses were captured verbatim, and subsequently translated. Content analysis was performed to identify their experience (message) with the paper-based and electronic data collection methods. Due to a small sample size, we reported the identities of the data collectors as DC in our results to maintain anonymity.

All data are available in a public, open access repository.^16^

## Results

**Figure 1:**
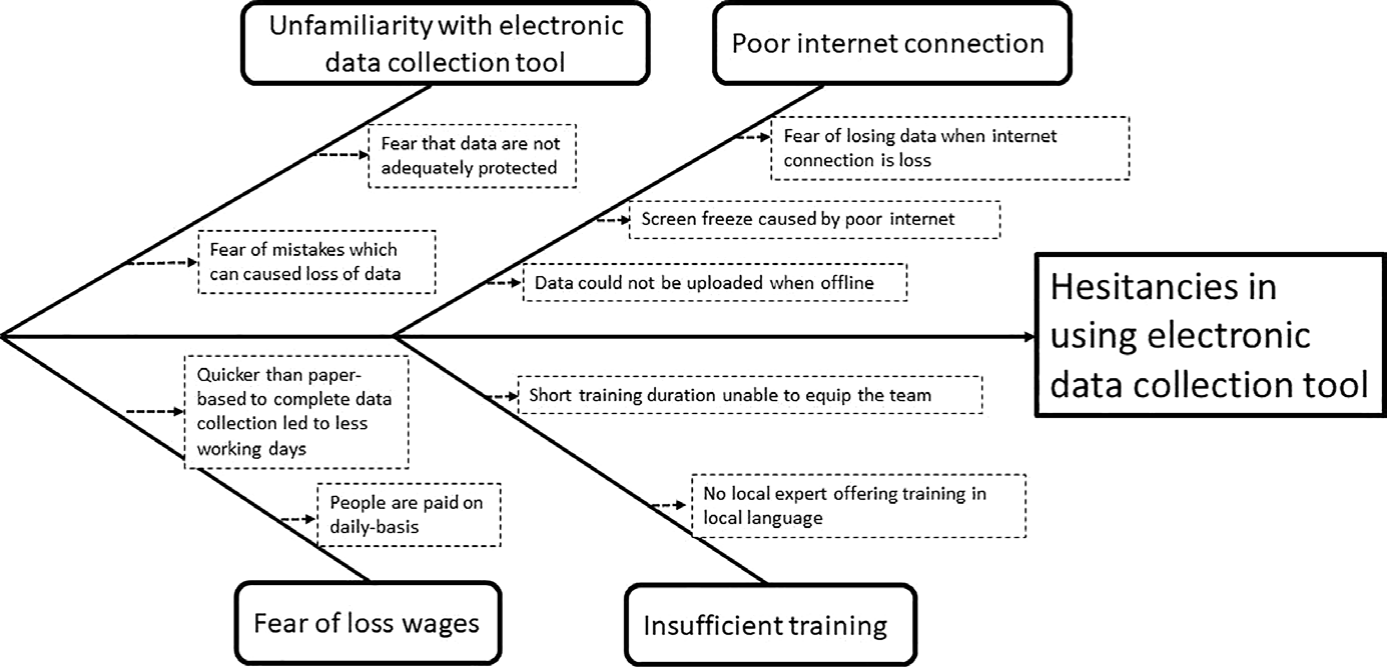
Fishbone analysis of the hesitancies in using electronic data collection tool.

The fishbone analysis (figure 1) shows why the local stakeholders hesitated to use electronic data collection tools for the WE-ZACE fieldwork.

- Concerns over poor internet connection that either causes the application not to work as planned (DC7; DC11) or data collected will be ‘lost’ (DC1). These concerns stemmed from negative experiences where studies delayed data collection time when faced with the application not functioning properly (DC3; DC5), inability to upload data to the central storage system offline (DC8; DC12) and experiencing screen freezes requiring the system to be rebooted multiple times (DC9; DC14). Some data were lost because the previous study used an internet-based data collection application.
- Concerns about insufficient training affecting data quality were raised, especially from older data collection members. They felt their lack of technology experience could lead to ‘slow progress and mistakes’ (DC2; DC4). Also, they felt that the ‘usual’ short training duration would prevent them from understanding the usage of the application well. Previous studies also used foreign trainers who did not converse in Kiswahili (the local language spoken in Zanzibar), and the training had to be translated from English to Swahili. This made the training inefficient and ineffective (DC1; DC2, DC3, DC4, DC8, DC12; DC13).
- Unfamiliarity with using electronic data collection tools has deterred them from using these tools. They felt that the electronic data collection system was too advanced for them (DC1), and they feared making mistakes (DC13). Some also raised concerns about the information shared with others by mistake because data were not adequately protected (DC8; DC9; DC13).
- The fear of loss wages was raised. From their perspective, paper-based data collection methods would take longer to complete data collection, and thus, would enable them to work more days for more wages. Using electronic data collection tools meant they could work faster and for a shorter period, leading to earning less (DC14).

### Data quality of baseline and endline

During baseline, there were 68 mandatory responses, with a potential 27 conditional responses for each participant. There were 283 participants, and based on their answers, we anticipated 20 351 responses. A total of 2611 errors (12.8%) were made, which included missing mandatory responses (n=1852, 70.9%), incorrect entries (n=503, 19.3%), illogical data (n=143, 5.5%) and missing conditional responses (n=113, 4.3%). During the endline, there were 54 mandatory responses, with a potential of 18 conditional responses for each participant. There were 215 participants included in the endline, and based on their answers, we anticipated 12 420 responses. There were two errors (0.02%) made, and both were incorrect spellings.

### Feedback on the data collection experience

#### Message 1: using a paper-based data collection tool during baseline was time-consuming, with plenty of errors

All the data collectors felt that collecting data during baseline was time-consuming because they needed to complete the forms in paper format and then capture them onto an electronic database. (‘During the baseline, we had to fill in the paper and then transfer the data into the laptop, thus consuming lots of time’. (DC11)) Many errors in the data collection forms. (‘There were so many errors made. You cannot read some of the handwriting. They were not legible. Some information did not make sense. Like, for a shortsighted woman, but (the) prescription was for longsighted’. (DC13)) Standardisation in recording was difficult to maintain. (*‘*We have already taught them to record visual acuity, pass or fail. Yet, some forms still recorded a Snellen’s fraction’. (DC3))

#### Message 2: the electronic data collection application was fast and simple

After completing the endline survey with the electronic data collection tool, the entire data collection team was impressed by how much time they saved. (‘use of electronic data collection tool made the process during endline saves time. The women could leave quickly without having them with us while we check for mistakes’. (DC13); ‘using a modern information collection system known as KOBO. We were quick and made the exercise go well’. (DC14)). The improved efficiency resulted from not needing to recapture data from paper onto a database. (‘Another thing that has been able to improve the collection of information is the use of technology where it has made it easier to save time where it has made me immediately after completing the collection of information to send it in the relevant system’. (DC13)) The electronic data collection system included skip logical, display logical, condition logical and prompt questions, which helped reduce the errors of inputting data to the minimum. (‘*…* while the endline survey used electronic devices only, this helps a lot to reduce human errors of wrong accounting as well as spelling errors’. (DC2); ‘Collecting data in electronic devices is much easier than paperwork during the first round (meaning the baseline). This is because the device has programmed the question with its logical sequence in skipping and pop-up messages to show that the value is unacceptable or is out of the range’. (DC14)) The ability to enter data offline and then upload it onto the central storage was also a feature received well by the team.

#### Message 3: there is a need to improve the electronic data collection methods and training

The team raised two important challenges that needed addressing. One, the team felt that using a device with a bigger storage capacity would be preferable. (‘By getting special tablet during the exercise and not on the phones because some phones have low capacity on internal storage’. DC8) Two, older team members knew the need to be trained better as they felt they ‘were not technology-savvy’ (DC3; DC4).

## Discussion

The present study was designed to address gaps in knowledge regarding electronic data collection tools in low-resource settings by analysing our fieldwork in Zanzibar. Our study demonstrates that electronic data collection is becoming increasingly feasible and significantly reduces response error in lower middle-income country research settings. Despite the increasing application of this tool, several challenges prevent an electronic data collection system’s widespread and routine use: poor programme functionality, inadequate training and unfamiliarity with electronic data collection.

Our study demonstrated that once the electronic data collection system had been properly implemented, errors in data were significantly reduced. This success is supported by existing data showing that electronic collection methods reduce survey error, expedite data transfer and prove to be teachable to people initially unfamiliar with the software.^7 8 17 18^ Existing data also revealed challenges similar to those our team faced: most notably, inadequate prior training on using electronic data collection tools resulted in unfamiliarity with the software and, thus, high response error when transferring from paper to electronic records.^7 8 17 18^

PAR to identify problems and prioritise and plan for data collection approaches. During the planning meeting, stakeholders in Zanzibar expressed several reservations about electronic data collection. These were programme functionality (crashing, internet connectivity, lost data), inadequate training (short training period, language gaps), unfamiliarity with electronic data collection, and fear of lost wages (due to potentially shorter data collection times). All these were valid concerns, and the history of inadequate internet access and cellular networks in rural areas is well documented.^19^ Fortunately, much of the infrastructure, such as internet connection, is improving to a point where it is now possible to adapt the electronic data collection tool to address these concerns.^12^

Among the challenges identified from this study, several recommendations for improvement exist. Stakeholders expressed concern about inadequate training for team members who were not ‘tech-savvy’ and low device storage, which prevented some from using the data collection application. These barriers could be overcome by increasing technology training for participants with limited experience and providing a higher storage device to enter data for participants with low storage.

We also identified several problems that could be sensitive and uncomfortable to address. For example, data collectors felt they would be penalised for working more efficiently using electronic data collection tool as they were paid daily for other projects. Because of this, most data collectors would choose to work slower to prevent loss of income. This phenomenon, known as ‘shirking’, happens as they see no incentive to engage and work faster.^20^ The team, therefore, decided to apply efficiency wages by paying a flat monthly rate, regardless if they completed their tasks earlier than planned. The stakeholders’ openness to discussing their concerns facilitated innovation and engagement to solve problems and work together effectively.

The data collectors experienced first-hand that data quality between paper-based and electronic-based data collection methods yielded significant differences, whereas during the baseline data collection phase, transferring from paper to electronic records demonstrated a high percentage of response error. Feedback from those collecting data provided valuable insights about the challenges and benefits of electronic data collection. Electronic data collection during baseline was time-consuming and fraught with errors. Some examples included illegible handwriting and illogical or incorrect data entry. Demonstrating these issues to our team and involving them in codeveloping an electronic data collection tool that addresses them has garnered their respect and instilled a sense of empowerment.

Once the electronic data collection system had been implemented, response errors decreased significantly. Users felt that electronic data collection was fast and simple because of reduced time to check mistakes, no need to recapture or transfer data from paper, and the use of skip logic to reduce errors. Mobile device-based tools and mobile phones have been increasingly used to collect and manage data in high-income countries and low/middle-income countries (LMICs)^21^ because of their great ability to collect high-quality data, user-friendliness and cost-effectiveness. However, even with a tremendous increase in access to the internet using wireless communication technologies, the upscale of its use in field research settings electronically in LMICs is still rare. The use PDSA method has helped our Zanzibar team to realise that electronic data collection is also feasible in their setting.

Our study’s findings are significant but warrant careful considerations for data collection in low-resource settings. One, we used a PAR methodology and PDSA model in this study instead of standard public health epidemiology methods, such as randomised trials. However, in our context, PAR has provided a platform to be more democratic, build capacity, encourage self-determination (reflection) and promote empowerment.^14^ Two, we used a shorter endline survey compared with the baseline survey. This may have introduced a lower probability for errors in the endline, but the proportion of errors was so insignificant and negligible during the endline that the survey length had limited effect on the results. Three, we assessed the efficiency of the electronic data collection tool, but not whether it influenced survey subjects’ perceptions of the researchers and the data’s trustworthiness, which warrants caution and investigation.

## Conclusion

The study, using PAR methods, found that Zanzibari stakeholders hesitated to adopt electronic data collection due to concerns about lost data, inadequate technology training, general unfamiliarity with technology and fear of lost wages. Collaboratively, we developed an electronic tool that significantly improved data quality, accuracy and efficiency compared with paper-based methods. However, addressing challenges like low device storage and training is crucial for optimal implementation and maximising benefits.

## Data Availability

Data are available in a public, open access repository.
